# Cardiovascular stability during arteriovenous extracorporeal therapy: a randomized controlled study in lambs with acute lung injury

**DOI:** 10.1186/cc2983

**Published:** 2004-10-28

**Authors:** Balagangadhar R Totapally, Jeffrey B Sussmane, Dan Torbati, Javier Gelvez, Harun Fakioglu, Yongming Mao, Jose L Olarte, Jack Wolfsdorf

**Affiliations:** 1 Miami Children's Hospital, Division of Critical Care Medicine, Miami, Florida, USA

**Keywords:** acute lung injury, arteriovenous extracorporeal membrane oxygenation, extracorporeal life support systems, hemodynamic stability, lamb

## Abstract

**Introduction:**

Clinical application of arteriovenous (AV) extracorporeal membrane oxygenation (ECMO) requires assessment of cardiovascular ability to respond adequately to the presence of an AV shunt in the face of acute lung injury (ALI). This ability may be age dependent and vary with the experimental model. We studied cardiovascular stability in a lamb model of severe ALI, comparing conventional mechanical ventilation (CMV) with AV-ECMO therapy.

**Methods:**

Seventeen lambs were anesthetized, tracheotomized, paralyzed, and ventilated to maintain normocapnia. Femoral and jugular veins, and femoral and carotid arteries were instrumented for the AV-ECMO circuit, systemic and pulmonary artery blood pressure monitoring, gas exchange, and cardiac output determination (thermodilution technique). A severe ALI (arterial oxygen tension/inspired fractional oxygen <200) was induced by lung lavage (repeated three times, each with 5 ml/kg saline) followed by tracheal instillation of 2.5 ml/kg of 0.1 N HCl. Lambs were consecutively assigned to CMV treatment (*n *= 8) or CMV plus AV-ECMO therapy using up to 15% of the cardiac output for the AV shunt flow during a 6-hour study period (*n *= 9). The outcome measures were the degree of inotropic and ventilator support needed to maintain hemodynamic stability and normocapnia, respectively.

**Results:**

Five of the nine lambs subjected to AV-ECMO therapy (56%) died before completion of the 6-hour study period, as compared with two out of eight lambs (25%) in the CMV group (*P *> 0.05; Fisher's exact test). Surviving and nonsurviving lambs in the AV-ECMO group, unlike the CMV group, required continuous volume expansion and inotropic support (*P *< 0.001; Fisher's exact test). Lambs in the AV-ECMO group were able to maintain normocapnia with a maximum of 30% reduction in the minute ventilation, as compared with the CMV group (*P *< 0.05).

**Conclusion:**

AV-ECMO therapy in lambs subjected to severe ALI requires continuous hemodynamic support to maintain cardiovascular stability and normocapnia, as compared with lambs receiving CMV support.

## Introduction

Neonatal, pediatric, and adult extracorporeal membrane oxygenation (ECMO), using venoarterial or venovenous modes, have been practised for over 3 decades [1-5]. These modes of ECMO are known to activate the inflammatory cascade [6,7], but the long-term cardiopulmonary outcome (10–15 years follow-up period) and neurodevelopmental outcome (at age 5 years) are relatively comparable to those in control individuals [8-10]. Patients who now receive ECMO therapy may also be different from patients treated in the 1980s and early 1990s because the alternative therapies have improved [11]. A search for safer modes of bypass therapy, including arteriovenous (AV)-ECMO, is warranted because of the cardiovascular and cerebral autoregulatory complications that are common during ECMO operations [12,13]. This new mode of ECMO therapy may have some advantages over conventional venoarterial ECMO or venovenous ECMO techniques because the AV-ECMO technique appears simpler and may involve fewer operational complications [14].

The first investigators to conduct AV-ECMO trials, Kolobow and coworkers [15] studied eight normal and conscious lambs (age 1–8 days) for periods up to 96 hours. They described reductions in hemoglobin concentrations during AV-ECMO therapy, showing some mild postmortem pulmonary pathology in a few cases. In a later study, those investigators [16] also designed a carbon dioxide membrane lung, which was used to reduce ventilation in spontaneously breathing or sedated animals subjected to controlled mechanical ventilation. They suggested that a carbon dioxide membrane lung could ideally be operated in an AV mode without using a pump.

The AV shunt of the AV-ECMO circuit requires adequate blood flow from the systemic circulation, which may require an increase in cardiac output (CO). Animal models of AV-ECMO without acute lung injury (ALI) show clinically acceptable cardiorespiratory stability [17-21], whereas models with ALI usually require inotropic and fluid support [13,22-26]. Conrad and coworkers [27], following a series of preclinical studies [14,23-25], evaluated the safety and efficacy of AV-ECMO therapy in a phase I clinical study. They treated eight patients (five males and three females, aged 21–67 years), who had acute respiratory failure and hypercapnia, with AV-ECMO over a 72-hour period. They found no significant changes in hemodynamic variables, whereas arterial carbon dioxide tension (PaCO_2_) was significantly reduced from 90.8 ± 7.5 mmHg to 51.8 ± 3.1 mmHg after 2 hours of AV-EMCO therapy [23]. At the same time, minute ventilation was reduced from a baseline of 6.92 ± 1.64 l/min to 3.00 ± 0.53 l/min.

AV-ECMO technique applied in the presence of ALI requires reasonable hemodynamic stability to permit an extracorporeal AV shunt sufficient for carbon dioxide clearance. Recently, we demonstrated that lambs with normal lungs are able to maintain effective CO and provide efficient ventilator support with a relatively moderate AV shunt of 15% [17]. The aim of the present study was to determine the cardiovascular support needed to maintain hemodynamic stability and the minute ventilation needed to maintain normocapnia in lambs subjected to severe ALI and treated with AV-ECMO (AV shunt flow of up to 15%) or conventional mechanical ventilation (CMV; AV shunt flow of 0%).

## Methods

### Surgical procedures

The experimental protocol for this study was approved by the Institutional Animal Care and Use Committee of the Mount Sinai Hospital Research Institute (Miami Beach, FL, USA). Seventeen lambs (aged 2–6 weeks, weight 3.6–12.7 Kg) and their ewes were transported to the laboratory at least 3 days before the experiments began. On the day of an experiment, an intravenous line was established, and anesthesia was induced (initial dose 50 mg/kg ketamine intravenously) and maintained throughout the experiment (5 mg/kg per hour intravenous ketamine). A 2% xylocaine solution was used to provide local anesthesia at the incision sites. A while after induction of anesthesia (30–45 min), a tracheotomy was performed and the lambs were connected to a ventilator (Adult Star Infrasonics, Inc., San Diego, CA, USA) at a fractional inspired oxygen (FiO_2_) of 1.0. Animals were then paralyzed with an intravenous bolus of 1.0 mg/kg vecuronium bromide, followed by 0.1 mg/kg per hour.

To establish an ECMO circuit, one internal jugular vein and one carotid artery were cannulated using neonatal ECMO catheters (Medtronic Bio-Medicus, Inc., Eden Prairie, MN, USA). A femoral vein was then cannulated using a 5 Fr Swan–Ganz catheter (Baxter Health Care Co., Critical Care Division, Irvine, CA, USA) for periodic measurement of CO employing the thermodilution technique (Oximetrix-3, CO Computer; Abbott Critical Care System, North Chicago, IL, USA) and for continuous recording of the mean pulmonary artery pressure (PAP). A femoral artery was cannulated for continuous monitoring of the mean arterial pressure (MAP; Datascope 2001; Datascope Co., Paramus, NJ, USA) as well as periodic blood sampling for gas analyses. A bolus of 200 U/kg heparin was administered intravenously, followed by a maintenance infusion of 200 U/kg per hour. Normothermia (38 ± 0.5°C) was maintained throughout the experiments. Lactated Ringer's solution (5 ml/kg per hour) was provided for fluid replacement.

### Procedures before injury

One hour after the completion of all invasive procedures, pre-ALI baselines were determined for all investigated variables. Arterial blood samples, corrected for body temperature, were measured using a blood gas analyzer (ABL-30; Radiometer, Copenhagen, Denmark). The same samples were used to measure arterial hemoglobin concentration and hemoglobin–oxygen saturation (Hb-O_2_) using a hemoximeter (OSM-3; Radiometer). CO was determined by the thermodilution technique using the indwelling Swan–Ganz catheter and a CO computer (Oximetrix-3; Abbot Critical Care System). Minute ventilation was measured using a neonatal respiratory monitor (Bicore Neonatal Respiratory System, Model CP-100; Bicore, Irvine, CA, USA). The ventilator tidal volume was set at 7 ml/kg body weight and positive end-expiratory pressure was set at 4 cmH_2_O. The peak inspiratory pressure was maintained below 30 cmH_2_O. Because arterial hypercapnia may affect the cardiovascular system [28], maximizing the ability of the heart to drive the AV shunt, we elected to maintain the PaCO_2 _between 30 and 45 mmHg, rather than allowing permissive hypercapnia to occur.

### Acute lung injury model

**Figure 1:**
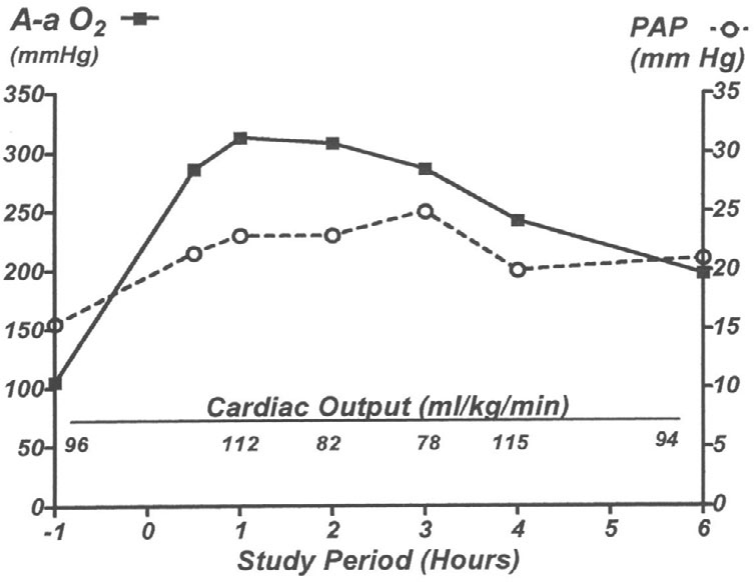
Changes in the average alveolar–arterial oxygen (A-a O_2_) gradient, pulmonary artery pressure (PAP), and cardiac output in two lambs after three separate lavages and intratracheal instillation of 2.5 ml/kg of 0.1 N HCl (fractional inspired oxygen 0.6). Time – 1 hour indicates baseline values before induction of acute lung injury (ALI). Data were periodically collected, starting 90 min after ALI procedures.

To establish a model of severe ALI, in a preliminary study we used the above surgical procedures without AV lines in two lambs. This was accomplished with three consecutive saline lavages (5 ml/kg saline for each). The third lung lavage was followed by an intratracheal instillation of a single dose of 2.5 ml/kg 0.1 N HCl. This procedure resulted in substantial increases in the alveolar–arterial oxygen gradient and an average 60% increase in PAP with relatively stable CO over an 8-hour study period (Fig. 1). Saline lavage followed by tracheal instillation of HCl was used in all animals administered CMV and AV-ECMO therapy. This combination may result in surfactant deficiency (caused by the saline lavage), and cellular injury and edema (caused by pulmonary exposure to acid).

### Post-acute lung injury procedures

In our ALI model significant arterial hypercapnia developed (data not presented), which was adjusted to relative normocapnia by changes in the respiratory frequency. Based on our preliminary results in the ALI model, we allowed a 90-min interval before determination of a postinjury baseline in order to stabilize gas exchange and hemodynamic parameters. During this recovery period, arterial blood gases were determined every 15 min. A postinjury baseline for all variables was then determined (time 0). At this stage, lambs were consecutively assigned either to continued CMV treatment or to AV-ECMO plus CMV therapy.

#### Group I

These lambs received continuous CMV support during a 6-hour study period with a closed AV shunt (*n *= 8). All hemodynamic, and arterial and venous mixed blood gas exchange variables were recorded every 2 hours. The oxygen content of both arterial and mixed venous blood was determined for calculation of oxygen consumption as a product of oxygen delivery (the difference between arterial oxygen content and mixed venous blood oxygen content) and CO (Fick's equation). Oxygen extraction was calculated using the differences between the measured values of arterial Hb-O_2 _and venous Hb-O_2 _saturation. After completion of the study period the lambs were euthanized by lethal dose of pentobarbital (100 mg/kg intravenously).

#### Group II

In this treatment group a set of baseline values were obtained during CMV with a closed AV shunt (*n *= 9). Subsequently, lambs were subjected to 6 hours of AV-ECMO plus CMV (AV-ECMO therapy) with a maximum AV shunt of 15% (calculated from CO measured during postinjury baseline). The AV-ECMO circuit was established using a hollow fiber oxygenator (Minimax; Medtronic, Inc. Minneapolis, MN, USA) primed with fresh maternal blood (150–200 ml). To test the efficiency of AV-ECMO as compared with that of CMV in terms of carbon dioxide clearance, we attempted to maintain relative normocapnia in both groups. This required changes in minute ventilation that were achieved by modifying the respiratory rate while maintaining peak inspiratory pressure below 30 cmH_2_O. To control the flow rate through the AV shunt, a clamp was placed on the arterial side of the AV-ECMO circuit and the flow was continuously measured (Medical Volume Flow Meter; Transonic Systems Inc., Ithaca, NY, USA).

Carbon dioxide clearance during an AV-ECMO operation is dependent on the gas flow through the oxygenator. The efficacy of carbon dioxide removal and oxygenation of the Minimax hollow fiber oxygenator were previously studied in our laboratory using 15% AV shunt during stepwise decreases in minute ventilation and oxygenation with gas flow of 1 l/min [17]. This gas flow was approximately four times the maximum blood flow through the AV shunt and maintained normocapnia with a 50% reduction in minute ventilation [17]. In the present study, the oxygenator's gas flow was kept constant at 1 l/min of 100% oxygen and was controlled by an in-line gas regulator (Servo pressure limited system; Hudson RCI, Temecula, CA, USA). To ensure proper performance of the oxygenators during AV-ECMO therapy, the post-oxygenator partial oxygen tension and partial carbon dioxide tension were measured at 2 and 6 hours during the study period.

### Resuscitative measures

The outcome measures in our study were the degree of cardiovascular support needed to maintain hemodynamic stability and the minute ventilation needed to maintain normocapnia during both CMV and AV-ECMO therapy. A number of resuscitative measures were used to maintain hemodynamic stability during both CMV and AV-ECMO trials. These included the following: boluses of 10 ml/kg per hour of lactated Ringer's solution, which were provided if MAP fell below 60 mmHg; infusion of dopamine (5 μg/kg per min) and epinephrine (adrenaline; 0.5–2 μg/kg per min) to maintain MAP above 60 mmHg, given if this MAP was not achieved with fluid resuscitation; and 1 mEq/kg sodium bicarbonate, which was given if the base excess was below –5 mmol/l despite institution of other resuscitative measures. The end-point for resuscitation was deemed to have occurred when all of the above measures failed and the MAP fell below 30 mmHg for a period of 15 min. This cutoff point was selected empirically because below this level of MAP the AV-ECMO animals could not maintain an AV shunt of over 5% of baseline CO.

### Statistical analyses

All values are expressed as mean ± standard deviation. Differences in specific variables after establishment of postsurgery baseline (60 min after completion of surgery) and post-ALI baseline (90 min after injury), both within the same group at different times and between the CMV and AV-ECMO groups, were evaluated using two-tailed unpaired t-tests. Data from the surviving lambs in the same group over the 6-hour study period were evaluated using analysis of variance (ANOVA), followed by Dunnett multiple comparisons test. For this analysis, we used the postinjury baselines in each variable as controls. Differences in each parameter among the surviving lambs in CMV and AV-ECMO groups and a group of nonsurvivors in the AV-ECMO category were evaluated using ANOVA, followed by Bonferroni multiple comparison test for comparable time periods. The use of resuscitative measures (lactated Ringer's, dopamine, epinephrine and bicarbonate) in all lambs after time zero and in the surviving lambs in the CMV and AV-ECMO groups, as well as mortality (death before completion of the 6-hour study period), were compared using Fisher's exact test. All resuscitative measures before baseline (time zero) were excluded from data analyses. *P *< 0.05 was considered statistically significant.

## Results

### Pre- and post-acute lung injury baselines

These data were collected in all animals (survivors and nonsurvivors) before assignment to the CMV or the AV-ECMO groups (Table 1). No significant differences were found between the preinjury values of lambs that were later randomized to CMV and AV-ECMO groups. After ALI, all lambs required significant increases in minute ventilation in order to achieve relative normocapnia (Table 1). Comparison of postinjury PaCO_2 _and pH between the two treatment groups revealed statistically significant differences in favor of the AV-ECMO group (Table 1). ALI created a arterial oxygen tension (PaO_2_)/FiO_2 _ratio of less than 200 (also representing PaO_2_) in both groups. After ALI, the PAP was significantly increased by approximately 50% in both groups. There were no significant differences in the postinjury baselines of MAP, PAP, and CO between the groups. The average body weight, measured before surgical procedures, was not significantly different between lambs consecutively assigned to CMV and those that were assigned to AV-ECMO (6.3 ± 1.7 kg versus 8.5 ± 2.8 kg, respectively). However, the four surviving lambs in the AV-ECMO group had significantly greater body weight than the five nonsurviving lambs (11.0 ± 2.2 kg versus 6.5 ± 1.3 kg; *P *< 0.05, by two-tailed unpaired t-test).

### Conventional mechanical ventilatory support versus arteriovenous extracorporeal membrane oxygenation therapy

**Figure 2:**
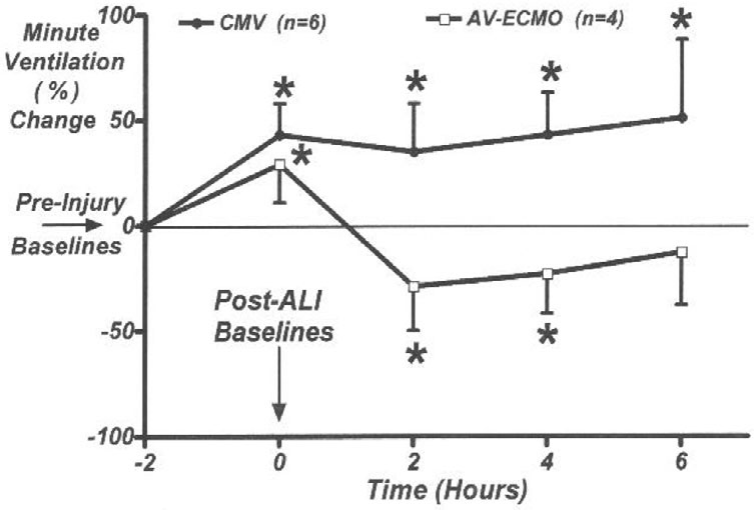
Comparisons between the minute ventilations (calculated per kg body weight) required to maintain normocapnia in lung-injured lambs subjected to conventional mechanical ventilation (CMV) or arteriovenous (AV)-extracorporeal membrane oxygenation (ECMO) with shunt flow of 15% of baseline cardiac output. Analysis of variance (ANOVA) followed by Dunnett multiple comparisons test was used to compare the preinjury level of minute ventilation in each group with subsequent measurements. ANOVA followed by Bonferroni test was used to compare CMV and AV-ECMO therapies at different time periods during the study period. Values are expressed as mean ± standard deviation. *P < 0.05. ALI, acute lung injury.

The data presented in Tables 2 and 3, and Figs 1 and 2 are from the surviving lambs only. Six out of eight lambs (75%) in the CMV group and four out of nine lambs (44%) in the AV-ECMO group survived the 6-hour study period after ALI. Three of the five nonsurviving lambs in the AV-ECMO group died within 45–90 min and two others died after 4 hours, despite a combination of resuscitative measures. On average, the surviving lambs in both groups had stable CO and MAP during the 6-hour study period (Tables 2 and 3). The four surviving lambs in the AV-ECMO group were able to maintain CO and MAP with varying degrees of hemodynamic support. This also allowed for a relatively stable AV shunt flow (14.8 ± 0.4% of the CO, measured at 0, 2, 4, and 6 hours) and a significant reduction of 25–30% in minute ventilation, as compared with the CMV group (Fig. 2).

**Figure 3:**
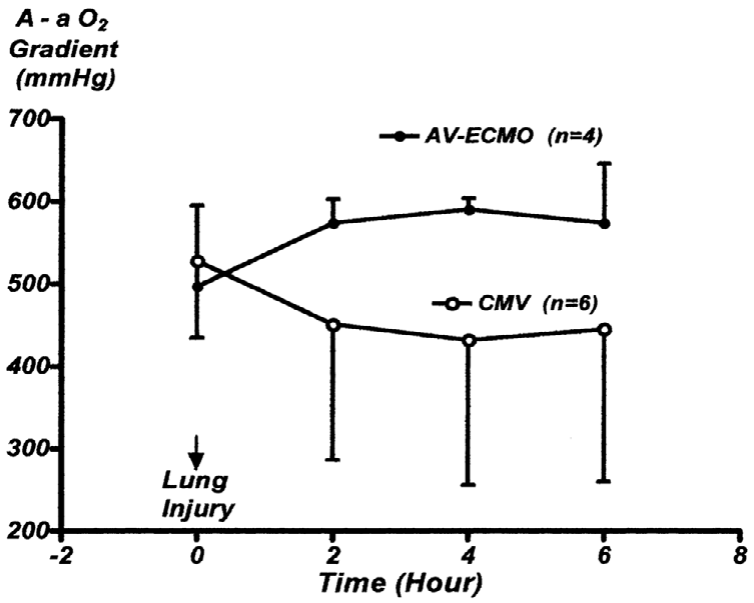
Changes in the alveolar–arterial oxygen (A-a O_2_) gradient in six lambs subjected to continued conventional mechanical ventilation (CMV) support and four lambs subjected to arteriovenous (AV)-extracorporeal membrane oxygenation (ECMO) therapy with a maximum shunt flow of 15%, up to 6 hours after establishment of acute lung injury (ALI). A-a O_2 _after ALI was consistently higher with AV-ECMO therapy than with CMV support. These differences became statistically significant at 4–6 hours, indicating higher deterioration in lung performance in the AV-ECMO group (repeated measures of analysis of variance followed by Dunnett multiple comparisons test, using the postinjury baseline in each group as controls).

There were no significant differences between the PaCO_2 _in CMV and AV-ECMO treated lambs during the study period, but the alveolar–arterial oxygen gradient was consistently higher in the AV-ECMO group (Fig. 3). The last measurements of MAP, PAP, and PaO_2_, which were obtained in four out of the five nonsurviving lambs in the AV-ECMO group, were 33.5 ± 9.3, 36.0 ± 6.3, and 53.7 ± 9.2 mmHg, respectively. These values were significantly lower than those recorded in the surviving lambs in either the AV-ECMO or the CMV group (Tables 2 and 3; ANOVA followed by Bonferroni multiple comparison test). Gas exchange of the oxygenators remained stable within the 6 hours of the study period. For example, the postoxygenator partial oxygen tension was 282 ± 8 mmHg and 282 ± 7 mmHg at 2 and 6 hours, respectively, and the postoxygenator partial carbon dioxide tension was 19.7 ± 5.1 mmHg and 21.0 ± 5.0 mmHg at 2 and 6 hours of AV-ECMO therapy.

### Hemodynamic stability

Analysis of the use of resuscitative measures as indicators of hemodynamic stability between the CMV and AV-ECMO groups revealed that significantly more lambs in the AV-ECMO group (including survivors and nonsurvivors) were resuscitated than in the CMV group (Table 4; *P *< 0.001, Fisher's exact test). However, there was no significant difference in 'mortality' between AV-ECMO and CMV groups within the 6-hour period of study (*P *> 0.05, Fisher's exact test).

## Discussion

The cardiovascular effects of AV-ECMO have been studied in adult and neonatal animal models [14-26]. It has been suggested that the resistance of the membrane oxygenator, hemodynamic stability, and the number, size and length of the conducting cannula, as well as the viscosity of the blood, will all affect the exogenous flow rate [22]. In the present study we utilized a low resistance membrane oxygenator, minimized the length of the conducting cannulae, and attempted to maintain MAP above 60 mmHg by using various resuscitative measures (Table 4). These measures in the AV-ECMO group failed to sustain hemodynamic stability in five out of nine lambs (56%), whereas the survivors (44%) were able to maintain normocapnia with a maximum of 30% reduction in minute ventilation over a 6-hour period of study (Fig. 2). The latter implies that AV-ECMO therapy, providing an AV shunt flow of up to 15% of the CO, may be able to reduce ventilator-induced lung injury in hypercapnic respiratory failure. However, in acute respiratory failure or acute respiratory distress syndrome with high intrapulmonary right-to-left shunt, extracorporeal blood flow in the range of 5–15% of CO may not be sufficient to provide adequate arterial oxygenation.

The reasons for the relatively poor performance of AV-ECMO therapy in our lamb model, as compared with the findings of studies conducted in adult animals [14,23,25], may be related to a number of factors. These possibilities are considered below.

First, differences between our model and other experimental models of ALI could account for differences between our findings and those of other studies. The present model may create a noncardiogenic pulmonary edema, which could be associated with loss of intravascular volume. Such conditions may require prolonged fluid and positive inotropic treatments to support a sufficient AV shunt flow. In comparison, Zwischenberger and coworkers [6,25] used an adult sheep model, in which acute respiratory distress syndrome was induced by smoke inhalation and 40% third degree burns. Sheep were then ventilated for 2 days before randomization to CMV and AV-ECMO (AV shunt of 11–14%) groups for a period of 7 days. There were no deaths in the AV-ECMO group (*n *= 8), as compared with only three survivors in the CMV group (*n *= 8). That model [6,25] demonstrates that perhaps a longer period of CMV support is needed to achieve relative cardiovascular stability before subjecting animals with severe ALI to the additional stress of an AV shunt.

How may a short recovery period after ALI affect hemodynamic stability during an AV-ECMO operation? ALI leads to the release of a variety of bioactive materials, including proinflammatory cytokines and reactive oxygen species [29]. The addition of an ECMO circuit to animals with ALI is known to stimulate the generation of inflammatory mediators, leading to further deterioration in cardiovascular function [6,7,16]. Zwischenberger and coworkers [6] studied the pathophysiology of ovine smoke inhalation lung injury after a relatively short recovery interval of 6 hours during both conventional ECMO therapy and CMV in female sheep. Those investigators demonstrated that animals treated with smoke and ECMO had significantly increased circulating thromboxane B_2 _levels and oxygen free radical activity, and a significant increase in lung wet:dry weight ratios. They suggested that an ECMO operation could potentiate the pathophysiology of smoke inhalation injury and lead to initial deterioration in native lung function [6]. Therefore, despite the simplicity of AV-ECMO procedures, as compared with conventional ECMO [14,30], it could be still subject to free radical generation because of presence of the membrane oxygenator. Thus, the addition of an AV shunt after ALI may further compromise the cardiovascular system.

A second factor that could account for the discrepancy between our findings and those of other investigators is that the AV shunt opening in our study led to a mortality rate in the smaller lambs, resulting in a difference between the body weights of the surviving lambs in two groups. This implies that smaller (and presumably younger) lambs with ALI could be more vulnerable to the presence of an AV shunt than relatively larger or older animals. Thus, studies concerning the safety and efficacy of neonatal AV-ECMO therapy should use animals with a narrow age range (1–7 days in lambs).

The third factor is whether the ALI in the CMV and AV-ECMO therapy groups was equal in severity. Whether the severity of ALI was different between the groups may be indirectly evaluated by comparing the indices of pre- and post-injury gas exchange. Our data indicate that pulmonary performance before starting AV-ECMO therapy was comparable with that observed in the CMV group (Table 1). The degree of lung injury was not significantly worsened during the 6-hour study period, as judged by lack of significant changes in alveolar–arterial oxygen gradient in the surviving lambs subjected to CMV or AV-ECMO therapy (Fig. 3).

### Study limitations

The outcome measures in this study were the degree of hemodynamic stability and the minute ventilation required to maintain relative normocapnia, while comparing CMV support with AV-ECMO therapy. Our study was not designed to evaluate mortality as an ultimate clinical outcome. A greater number of lambs would have been required to demonstrate significant differences in mortality between the CMV and AV-ECMO groups. However, the more than 50% mortality rate in the AV-ECMO group may raise questions about the clinical and/or statistical significance of our findings. Technically, we failed to use a narrow range of age and body weight in our lambs. However, the average body weights in lambs consecutively randomized to CMV support and AV-ECMO therapy were not significantly different (Table 1).

## Conclusion

Our study indicates that cardiovascular support is required to maintain hemodynamic stability during application of AV-ECMO therapy in lambs with severe ALI. In this model, AV-ECMO therapy with continuous cardiovascular support and an AV shunt flow of 15% of CO can provide a maximum 30% reduction in minute ventilation. We suggest that AV-ECMO with cardiovascular support [30] could be suitable for use in ALI of mild severity, in which permissive hypercapnia is not an acceptable treatment [28,31].

## Key messages

• 	Continuous hemodynamic support is required during AV-ECMO in lambs subjected to severe ALI.

• 	By using a shunt flow of up to 15% of CO, AV extracorporeal therapy in lambs with severe ALI can reduce minute ventilation by 25–30%.

• 	Neonatal patients with severe ALI and hemodynamic instability may not be suitable candidates for AV-EMCO therapy.

## Abbreviations

ALI = acute lung injury; ANOVA = analysis of variance; AV = arteriovenous; ECMO = extracorporeal membrane oxygenation; CMV = conventional mechanical ventilation; CO = cardiac output; FiO_2 _= fractional inspired oxygen; Hb-O_2 _= hemoglobin–oxygen saturation; MAP = mean arterial pressure; PaCO_2 _= arterial carbon dioxide tension; PaO_2 _= arterial oxygen tension; PAP = pulmonary artery pressure.

## Competing interests

The author(s) declare that they have no competing interests.

## Author's contributions

BRT, JBS and DT completed the proposal writing and experimental design. DT and BRT participated in research coordination, data analysis and presentation. JG, HF, YM, and JLO conducted all experimental aspects of the study. BRT, DT, JBS, and JW prepared the manuscript.

## Figures and Tables

**Table 1:** Comparison of cardiorespiratory variables before and after induction of lung injury

	CMV (*n *= 8)		AV-ECMO (*n *= 9)	
	
Variables	Pre-injury	Post-injury	Pre-injury	Post-injury
Minute volume (ml/kg/min)	408 ± 79	640 ± 144*	382 ± 109	561 ± 187*
PaO_2 _(mmHg)	389 ± 128	113 ± 85***	393 ± 131	179 ± 86***
PaCO_2 _(mmHg)	37.1 ± 5.1	40.9 ± 3.1	36.7 ± 1.9	35.1 ± 3^†^
Arterial pH	7.311 ± 0.05	7.263 ± 0.04	7.349 ± 0.05	7.344 ± 0.03^††^
HCO_3_^- ^(mmol/l)	17.7 ± 3.2	17.4 ± 1.7	19.5 ± 2.8	18.3 ± 2.1
Arterial Hb-O_2 _(%)	99.8 ± 0.3	89.6 ± 11	99.8 ± 0.3	95.1 ± 11
O_2_-extraction (%)	28.0 ± 5.7	35.9 ± 4.9*	24.7 ± 8.1	35.7 ± 9.3*
Hb (g/dl)	9.4 ± 1.5	10.3 ± 2.0	8.1 ± 2.0	8.7 ± 2.3
MAP (mmHg)	84.2 ± 12.1	88.0 ± 10.9	96.7 ± 9.0	92.0 ± 14
PAP (mmHg)	13.1 ± 4.1	20.1 ± 5.7	14.5 ± 5.4	21.5 ± 4.9*
CO (ml/kg per min)	185 ± 23	164 ± 53	181 ± 69	173 ± 56
VO_2 _(ml/kg per min)	5.7 ± 2.2	8.7 ± 2.7*	5.4 ± 1.5	7.8 ± 2.5*
Body weight (kg)				
All lambs	6.3 ± 1.7	-	8.5 ± 2.8	-
Surviving lambs	-	6.5 ± 1.3	-	11.0 ± 2.2^†^
Range	3.6–9.2	-	5.0–12.7	-

Comparison of cardiorespiratory variables before and after induction of lung injury in surviving and nonsurviving lambs subjected to conventional mechanical ventilation (CMV) or arteriovenous (AV)-extracorporeal membrane oxygenation (ECMO) therapy. Values are expressed as mean ± standard deviation. *P < 0.05, **P < 0.01, ***P < 0.001, pre-injury baseline versus post-injury baseline in the same group. ^†^*P *< 0.05, ^††^*P *< 0.01, pre-injury or post-injury baselines: CMV versus AV-ECMO. CO, cardiac output; Hb-O_2_, hemoglobin–oxygen saturation; MAP, mean arterial pressure; PaCO_2_, arterial carbon dioxide tension; PaO_2_, arterial oxygen tension; PAP, pulmonary artery pressure; VO_2_, oxygen consumption.

**Table 2:** Hemodynamics and oxygen consumption

	Study period (hours after establishment of acute lung injury)			
	
Variables	0 (baseline)	2	4	6
Minute ventilation (ml/kg per min)				
CMV	624 ± 189	550 ± 152	590 ± 151	608 ± 192
AV-ECMO	397 ± 96	214 ± 83^†^*	222 ± 128^†^*	256 ± 155^†^*
MAP (mmHg)				
CMV	89.6 ± 12.2	81.5 ± 10.1	83.8 ± 17.0	82.5 ± 9.0
AV-ECMO	92.5 ± 10.6	90.0 ± 16.2	73.5 ± 25.0	74.5 ± 40
PAP (mmHg)				
CMV	20.8 ± 6.4	23.1 ± 7.7	22.6 ± 3.7	18.6 ± 6.8
AV-ECMO	19.0 ± 5.7	24.7 ± 7.5*	26.2 ± 7.8*	26.2 ± 9.3*
Cardiac output (ml/kg per min)				
CMV	164 ± 45	165 ± 28	189 ± 62	194 ± 54
AV-ECMO	144 ± 56	177 ± 30	143 ± 47	184 ± 54
Oxygen consumption (ml/kg per min)				
CMV	9.6 ± 2.4	9.2 ± 1.5	10.1 ± 3.4	8.9 ± 3.7
AV-ECMO	7.1 ± 1.7	9.7 ± 2.3	8.1 ± 1.3	8.2 ± 3.7

Hemodynamics and oxygen consumption in lung injured lambs (surviving) supported by conventional mechanical ventilation (CMV; *n *= 6) or arteriovenous (AV)-extracorporeal membrane oxygenation (ECMO; *n *= 4) during a 6-hour period of study. Values are expressed as mean ± standard deviation. *P < 0.05, baseline (time 0) versus 2, 4, and 6 hours of study by repeated measures analysis of variance (ANOVA) followed by Dunnett multiple comparisons test. ^†^*P *< 0.05, CMV versus AV-ECMO groups; ANOVA followed by Bonferoni multiple comparisons test.

**Table 3:** Gas exchage variables

	Study period (hours after establishment of acute lung injury)			
	
Variable	0 (baseline)	2	4	6
PaO_2 _(mmHg)				
CMV	131 ± 90	207 ± 171	231 ± 175	221 ± 189
AV-ECMO	174 ± 100	95 ± 29	77 ± 17*	94 ± 76
PaCO_2 _(mmHg)				
CMV	41.3 ± 3.1	43.4 ± 8.3	38.9 ± 10.3	37.0 ± 7.1
AV-ECMO	34.8 ± 2.3**	35.1 ± 8.5	37.1 ± 7.8	37.8 ± 5.7
pH				
CMV	7.263 ± 0.05	7.237 ± 0.05	7.286 ± 0.10	7.289 ± 0.08
AV-ECMO	7.322 ± 0.04*	7.277 ± 0.15	7.235 ± 0.10	7.207 ± 0.14
HCO_3_^- ^(mmol/l)				
CMV	17.0 ± 3.1	16.8 ± 3.2	16.7 ± 2.7	15.9 ± 3.3
AV-ECMO	17.2 ± 2.4	15.7 ± 2.3	15.7 ± 5.4	14.6 ± 4.6
Arterial Hb-O_2 _(%)				
CMV	91.8 ± 8.1	89.2 ± 14.9	94.0 ± 4.9	88.9 ± 18.4
AV-ECMO	90.7 ± 16.5	89.0 ± 9.9	84.4 ± 7.0	74.0 ± 24.8
O_2 _extraction (%)				
CMV	42.4 ± 15.6	37.6 ± 9.2	47.2 ± 11.5	41.2 ± 6.9
AV-ECMO	41.6 ± 11.9	37.3 ± 14.2	49.3 ± 21.0	43.3 ± 21.7

Gas exchage variables in lung injured lambs (surviving) supported by conventional mechanical ventilation (CMV; *n *= 6) or arteriovenous (AV)-extracorporeal membrane oxygenation (ECMO; *n *= 4) during a 6-hour period of study. No significant differences were found when comparing baselines (time 0) with 2, 4, and 6 hours of study by repeated measures analysis of variance (ANOVA) followed by Dunnett multiple comparisons test. Values are expressed as mean ± standard deviation. *P < 0.05, **P < 0.01, CMV versus AV-ECMO group; ANOVA followed by Bonferroni multiple comparisons test. Hb-O_2_, hemoglobin–oxygen saturation; PaCO_2_, arterial carbon dioxide tension; PaO_2_, arterial oxygen tension.

**Table 4:** Numbers of lambs undergoing various resuscitative measures

Type of support	Group		*P*^a^
		
	CMV (*n *= 8)	AV-ECMO (*n *= 9)	
Lactated Ringer's (10 ml/kg)	2	8	0.015
Epinephrine (0.5–2 μg/kg per min)	1	6	0.049
Dopamine (5 μg/kg per min)	1	6	0.049
Bicarbonate (1 mEq/kg bolus)	1	6	0.049
Surviving/nonsurviving	6/2	4/5	0.333
Total number of resuscitative measures in surviving lambs	2 (*n *= 6)	12 (*n *= 4)	0.001
Cause of death	Prolonged hypotension with MAP <30 mmHg	Prolonged hypotension with MAP <30 mmHg and AV shunt <5% of CO	

Comparison of various resuscitative measures after acute lung injury in surviving and nonsurviving lambs subjected to conventional mechanical ventilation (CMV) with closed arteriovenous (AV) shunt or CMV with AV-extracorporeal membrane oxygenation (ECMO) using up to 15% AV shunt. ^a^*P *values derived using Fisher's exact test. CO, cardfiac output; MAP, mean arterial pressure.
